# Comment on “Feasibility of a New Cuffless Device for Ambulatory Blood Pressure Measurement in Patients With Hypertension: Mixed Methods Study”

**DOI:** 10.2196/15952

**Published:** 2020-04-22

**Authors:** Noud van Helmond, Wim J Jongejan, Robert A Hirsh

**Affiliations:** 1 Department of Anesthesiology Cooper Medical School of Rowan University Cooper University Health Care Camden, NJ United States

**Keywords:** blood pressure measurement, cuffless, hypertension

We read the article from Ogink and colleagues [1] describing the use of cuffless blood pressure (BP) measurement for home BP measurement. We applaud the authors’ intention to study the relevance of cuffless BP measurement in real-world conditions and have a few comments and questions that may improve our understanding of this study’s results.

First, we previously commented [2] on a prior study performed by the same group [3]. We highlighted problems with the Checkme’s accuracy and its marketing in the United States as a systolic BP monitor without the required Food and Drug Administration approval. We are surprised that the authors refer to the prior study performed by Schoot et al [3] as promising in terms of European Society of Hypertension accuracy standards and by their repeated erroneous claim that the Checkme has Food and Drug Administration approval for measurements of systolic BP [1], after acknowledging the Checkme’s shortcomings related to accuracy and regulatory approval in a response to our letter [4]. It is important to note that the referenced CE (Conformitè Europëenne) certification constitutes conformity with electromagnetic safety standards but is not a certification for demonstrated accuracy and precision of systolic BP measurements.

Second, in the referenced study, the weak correlation (R=0.47) between paired home cuff systolic BP measurements and Checkme measurements, as well as a large absolute difference between these measurements (eg, 44% measurements differed by >10 mm Hg) confirms the inaccuracy of individual Checkme systolic BP measurements. Over the course of 3 weeks, the average of all twice-daily, duplicate, Checkme measurements (84 total measurements in each subject) correlated better (R=0.75) with the average of all once-weekly, duplicate home cuff BP measurements over 3 weeks (6 total measurements in each subject), but these means still varied by 5-15 mm Hg for 64% of measurements. The authors suggest that the average of a large number of Checkme measurements can be used by physicians to adjust medication. Considering that the mean treatment response of antihypertensive medications lies in the 5-15 mm Hg range for systolic BP [5], how do the authors envision Checkme measurements to be used in clinical care?

Finally, a major reported advantage of the Checkme is its user-friendliness as assessed by semistructured interviews. We note that individuals were excluded if they were not able to perform the Checkme measurement correctly after 20-40 minutes of instruction. Can the authors please share how many patients were not enrolled after failure to perform the measurement after instruction? Further, we wonder if the required measurement frequency of Checkme as compared to conventional measurements (14 times as much) was assessed in the structured interviews?

## Abbreviations

BP: blood pressure
CE: Conformitè Europëenne
